# District-level strategies to control the HIV epidemic in Zimbabwe: a practical example of precision public health

**DOI:** 10.1186/s13104-020-05234-8

**Published:** 2020-08-26

**Authors:** Richard Makurumidze, Tom Decroo, Lutgarde Lynen, Zororo Kudzaishe Chinwadzimba, Wim Van Damme, James Hakim, Simbarashe Rusakaniko

**Affiliations:** 1 College of Health Sciences, University of Zimbabwe, Harare, Zimbabwe; 2 Institute of Tropical Medicine, Antwerp, Belgium; 3 Research Foundation of Flanders, Brussels, Belgium; 4 Gerontology, Faculty of Medicine & Pharmacy, Free University of Brussels (VUB), Brussels, Belgium; 5 Ministry of Health and Child Care, Geographic Information System Department, Harare, Zimbabwe

**Keywords:** HIV testing, Linkage to care, ART coverage, 90-90-90 targets, Implementation, Precision public health, Zimbabwe

## Abstract

**Objective:**

We conducted a descriptive cross-sectional study using survey and programme data to assess district-level performance along the HIV care cascade (HIV testing target achievement, linkage to ART and ART coverage) in order to formulate district-specific recommendations, taking into consideration prevalence and yield of testing.

**Results:**

Data from 60 districts were analysed. Forty-eight districts (80.0%) surpassed 90% of their 2018 HIV testing targets. Linkage to ART was less than 90% in 40 districts (83.3%). Thirty districts (50.0%) had ART coverage above 90%. Of the 30 districts with suboptimal (< 90%) ART coverage, 18 districts had achieved high HIV testing target but with suboptimal linkage to ART, 6 had achieved high HIV testing targets and high linkage to ART, 4 had both suboptimal HIV testing target achievement and linkage to ART and 2 had suboptimal HIV testing target achievement and high linkage to ART. Priority should be given to districts with suboptimal ART coverage. Remediation strategies should be tailored to address the poorly performing stage of the cascade in each of the districts.

## Introduction

In 2019, Zimbabwe had about 1.2 million [95% confidence interval (CI): 1.1–1.4 million] people living with HIV (PLHIV) [1]. The number of new HIV infections decreased by 35.3%, from 62 000 [95% CI 45 000–83 000] to 38 000 [95% CI 28 000–51 000] between 2010 and 2018. HIV-related deaths also decreased by 42.6%, from 54,000 [95% CI 43,000–68,000] to 22,000 [95% CI 17,000–27 000] during the same period [1]. The Zimbabwe Population-Based HIV Impact Assessment (ZIMPHIA) survey, conducted in 2015–2016 to assess progress towards the UNAIDS 90-90-90 targets [2], showed that 74.2% of all PLHIV reported knowing their HIV status, of whom 86.8% were on antiretroviral therapy (ART) and 86.5% of those on ART were virally suppressed [3].

Most countries show national data, without evidence on whether this mirrors the data at sub-national levels. Hence, most national programmes, including those in Zimbabwe, do not provide recommendations adapted for district-level indicators. At the same time, donors call for targeted and cost-effective strategies, due to dwindling funds [4, 5]. Advances in information technology provide an opportunity for data-driven health interventions. In our study, we combined mapping data on HIV prevalence [6] with data from the Zimbabwe National ART Program 2018. We studied the district-level performance along the HIV care cascade (HIV testing target achievement, linkage to ART and ART coverage) and formulated district-level recommendations considering HIV prevalence and HIV testing yield.

## Main text

### Methods

We conducted a descriptive cross-sectional study and retrieved the district-level HIV prevalence from the mapping study [6].

For each district, the number of people tested and the number tested positive and initiated on ART in 2018, and the total number of clients on ART at the end of 2018 were obtained from the Ministry of Health and Child Care (MoHCC), District Health Information System 2 (DHIS 2) [7]. Permission was obtained from the relevant authorities.

To estimate the district-level HIV testing achievement in 2018, we first calculated the district-level targets. We calculated the percentage of PLHIV in every district by dividing the estimated number of PLHIV in a district with the estimated total number of PLHIV in the country in 2018. We then use this percentage to distribute the overall national HIV testing target for 2018 to the districts [8, 9]. Second, we calculated the testing coverage by dividing the number of tests conducted with the district target (number of tests proposed by each district) [10]. The HIV testing yield was calculated by dividing the number of positive tests with the number of tests conducted per district.

Linkage to ART was estimated by dividing the number of patients that were started on ART in 2018 with the number of positive tests in the same year (with the assumption that under the World Health Organization’s HIV “Treat All” recommendations, all those tested positive had been put on ART [11]). The ART coverage was estimated by dividing the number of patients reported as active on ART at the end of 2018 in a district with the total number of PLHIV in that district.

HIV testing target achievement, linkage to ART and ART coverage were categorised into low (< 70%), medium (70–90%) and high (> 90%) and the district-level categorisation was visualized on maps using geographic information systems (GIS).

We also described district-level performance along the HIV cascade for those districts with suboptimal (< 90%) ART coverage. These were grouped as follows: 1. Suboptimal HIV testing target achievement/suboptimal linkage to ART, 2. Suboptimal HIV testing target achievement/high linkage to ART, 3. High HIV testing target achievement/suboptimal linkage to ART and 4. High HIV testing target achievement/high linkage to ART.

### Results

**Fig. 1:**
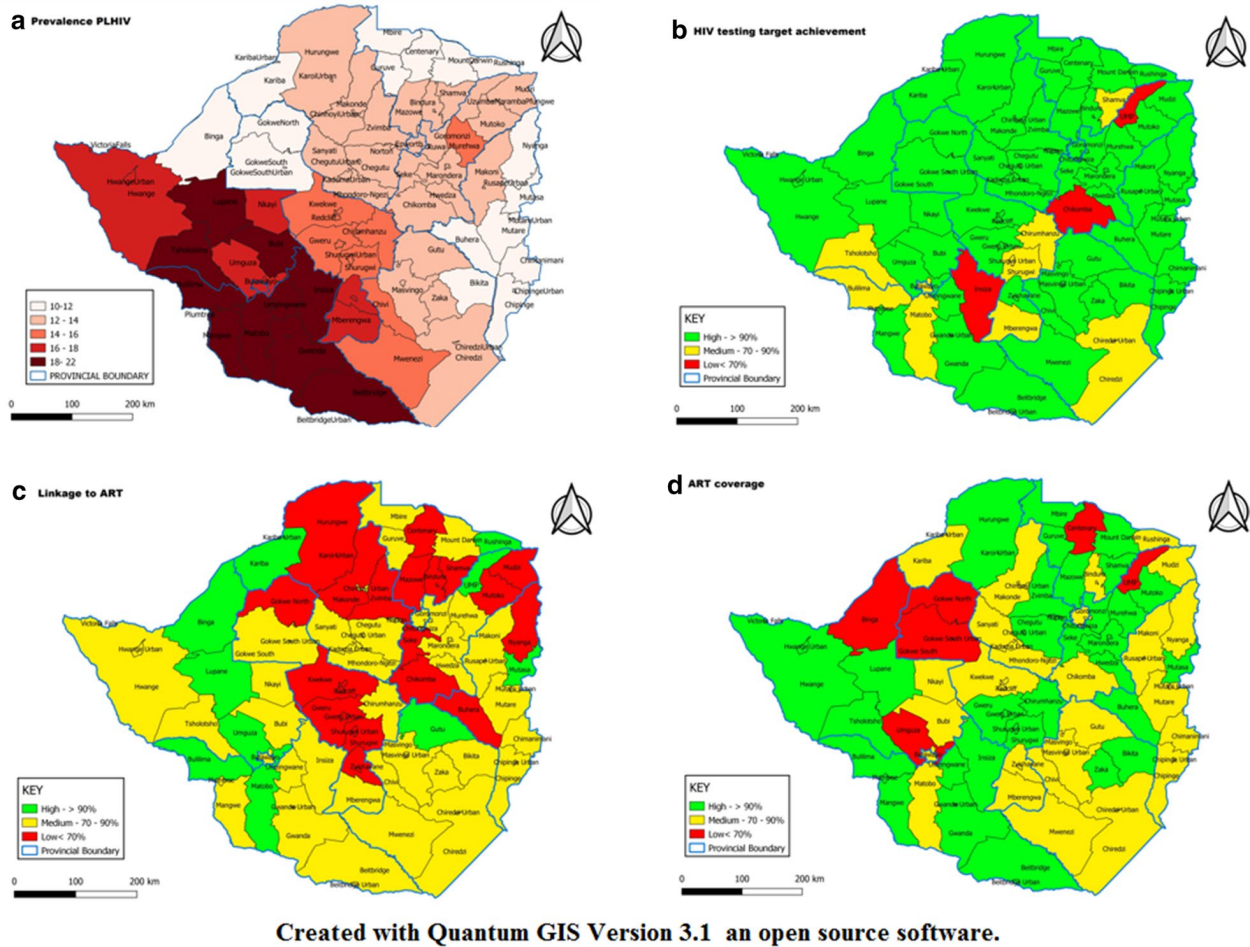
Maps showing; **a** HIV prevalence, **b** HIV testing target achievement, **c** linkage to ART, **d** ART coverage

We included all the districts of Zimbabwe in the analysis. District-level categorization of HIV prevalence, HIV testing target achievement, linkage to ART and ART coverage are shown in Fig. 1.

The highest prevalence was in Bubi District (21.5%) followed by Bulilima District (20.8%) and Tsholotsho District (20.7%). Forty-eight districts (80.0%) surpassed 90% of their 2018 HIV testing target. Only three reported a low (< 70%) testing coverage: Insiza (69.2%), Chikomba (62.7%) and Umzingwane (62.5%). A total of 29 districts (48.3%) had an HIV testing yield between 5.0 and 7.5% (Additional file 1: Figure S1). Linkage to ART was less than 90% in 40 districts (83.3%).

Thirty districts (50.0%) had high ART coverage (> 90%). Of the 30 districts with suboptimal coverage, 24 districts (40.0%) had medium coverage (70–90%), while the ART coverage was low (< 70%) in 6 districts (10.0%): Gokwe North (39.4%), Gokwe South (44.4%), Umguza (49.7%), Centenary (61.0%), Uzumba-Maramba-Pfungwe (63.0%) and Binga (68.0%).

Table 1 shows the district-level performance along the HIV cascade among 30 districts with suboptimal ART coverage (< 90%). Of the 30 districts, 4 had both suboptimal HIV testing target achievement and linkage to ART, 18 had high HIV testing target achievement but suboptimal linkage to ART, 6 had high HIV testing target achievement and high linkage to ART and 2 had suboptimal HIV testing coverage but high linkage to ART.

**Table 1:** HIV cascade for 30 districts with suboptimal (< 90%) ART coverage in 2018, Zimbabwe

District	ART Coverage (%)	HIV target testing achievement (%)	Linkage to ART (%)	Yield (%)	Prevalence (%)
Suboptimal HIV testing target achievement and suboptimal linkage to ART					
Chikomba	77	63	54	7	13
Mberengwa	77	89	71	6	17
Chiredzi	80	89	81	6	13
Bulawayo	83	73	77	8	16
High HIV testing target achievement and suboptimal linkage to ART					
Gokwe North	39	125	63	4	10
Gokwe South	44	129	71	4	12
Centenary	61	119	55	6	12
Bubi	70	131	83	4	22
Bindura	75	140	29	15	14
Nyanga	75	123	35	7	11
Zvishavane	75	117	59	8	18
Harare	78	110	75	7	13
Kadoma	78	358	72	7	13
Nkayi	78	179	86	3	17
Kwekwe	79	115	67	8	14
Mudzi	81	96	43	6	12
Makonde	82	189	69	6	13
Mutare	82	206	80	4	11
Chipinge	85	123	81	5	11
Mwenezi	86	122	76	7	15
Chimanimani	88	155	74	4	11
Masvingo	88	142	74	7	14
High HIV testing target achievement and high linkage to ART					
Umguza	50	94	96	5	18
Binga	68	122	121	2	12
Rushinga	75	137	101	2	11
Gutu	77	132	100	3	13
Makoni	87	151	90	4	12
Kariba	89	184	100	5	12
Suboptimal HIV testing target achievement and high linkage to ART					
UMP	63	63	110	7	12
Matobo	76	80	105	5	19

### Discussion

In Zimbabwe, half of the districts had high (> 90%) ART coverage, and thus had achieved the second target of the UNAIDS 90-90-90 targets [2]. However, substantial within-country variations in terms of HIV prevalence, HIV testing coverage, HIV testing yield, linkage to ART and ART coverage were observed. We have proposed four categories of districts according to gaps along the HIV cascade, for which district-specific recommendations can be formulated.

#### Suboptimal HIV testing target achievement and suboptimal linkage to ART

In the four districts with both suboptimal HIV testing achievement and linkage to ART, both the HIV prevalence and the HIV testing yield were above average, highlighting an important unmet need for HIV testing services. Therefore, HIV testing services should become more widely available. It has been seen that widespread door-to-door HIV testing substantially increases the acceptance of HIV testing and should be considered as a priority [12]. In addition, strategies linking those who tested positive to ART should be strengthened (elaborated below).

#### High testing target achievement and suboptimal linkage to ART

Eighteen districts with suboptimal ART coverage that achieved their HIV testing target but with suboptimal linkage to ART should assess if strategies known to improve linkage to care and ART initiation are in place. These strategies include same-day ART initiation, community (home-based) ART initiation, decentralisation of ART services to the primary health care level and integration of HIV care in other health care services [13–17]. Linkage to ART should be regularly assessed at the health facility level. Those who are diagnosed with HIV but have not been started on ART should be tracked. Consent for tracking should be incorporated into the testing strategy [18]. Patients diagnosed at higher-level referral health facilities should be initiated on ART before down referral to lower-level health facilities for follow up, with tracking of arrival after referral [19]. Some districts may have specific challenges. Poor linkage to ART around Kwekwe and the surrounding districts might be explained by the presence of illegal artisanal miners in the region. Illegal artisanal miners are highly mobile, and strategies to link and retain these highly mobile populations should be identified [20, 21]. Also, in districts bordering Zambia and Mozambique (Centenary, Mudzi and Nyanga), the poor linkage may be due to patients crossing the border as a result of the economic challenges in Zimbabwe. The Southern African Development Community (SADC) HIV and AIDS Cross Border Initiative should be fully implemented to enable the provision of care and tracking of such patients [22–24].

#### High testing target achievement and high linkage to ART

Six districts with suboptimal ART coverage achieved their HIV testing targets and had high linkage to ART. In such a scenario, other indicators may assist in the formulation of a district-specific strategy. If the prevalence and HIV testing yield are lower than average, HIV testing may need to be delivered in a more targeted manner. Health facility-based strategies in combination with community testing in high-risk groups may be most efficient. Health facility-based strategies that have worked elsewhere include index case testing, targeting sexual partners and HIV-exposed infants and intensified provider-initiated testing and counselling (IPITC) [25–31]. Community testing should prioritise subgroups with a higher prevalence. Key populations and hotspots identified from a mapping exercise conducted in Zimbabwe may guide programming [32]. Social network testing, using peer educators, can be a useful tool to reach some of the key populations [30].

#### Suboptimal HIV testing target achievement and high linkage to ART

There were two districts with suboptimal ART coverage that had suboptimal HIV testing target achievement but with high linkage to ART. In both, the HIV prevalence and testing yield were higher than average. Hence, HIV testing strategies recommended for high prevalence settings, discussed above, should be considered.

In conclusion, there is substantial within-country variation in terms of HIV prevalence, HIV testing target achievement, HIV testing yield, linkage to ART and ART coverage. Hence, a “one size fits all” approach is unlikely to result in achieving the next UNAIDS 95-95-95 targets by the end of 2030. District-level mapping of uncovered needs and gaps along the HIV cascade of care is needed, particularly for districts with suboptimal ART coverage.

## Limitations

Our study is among the first to assess the performance across the HIV care cascade at the sub-national level. To validate our findings, we compared data from a variety of sources. However, there are limitations due to the cross-sectional design of our study. Crossover of patients between prior or later years with 2018 may have occurred, for instance, when patients diagnosed in 2017 started ART in 2018, and those diagnosed in 2018 started ART in 2019. The district HIV testing targets were calculated by using the percentage of the number of PLHIV per district to distribute the national HIV testing target without considering the context and performance of each district cascade of care. The MoHCC have since started estimating district yearly HIV testing targets by taking into consideration the context and district-specific parameters. Moreover, district-level data on the third “90” from the UNAIDS 90-90-90 targets, i.e. viral suppression, was unavailable. Previous studies showed that the retention on ART was high, while access to viral load monitoring and viral load suppression remained suboptimal [33–35].

## Supplementary information

**Additional file 1: Figure S1.** Yield per district among HIV tests performed in 2018 in Zimbabwe.

## Data Availability

Zimbabwe data on HIV prevalence and the number of PLHIV is publicly available at http://ghdx.healthdata.org/ihme-data/africa-hiv-prevalence-geospatialestimates-2000–2017. The data from the Ministry of Health and Child Care on the number on ART per district by the end of 2018 is not available in the public domain. Anyone interested in using the data for scientific purpose is free to request permission from the Director of the AIDS and TB Program, AIDS and TB Unit, Ministry of Health and Child Care, Government of Zimbabwe, 2nd Floor, Mukwati Building, Harare, Zimbabwe. Email: atp.director@ymail.com

## Abbreviations

CI: Confidence interval
MoHCC: Ministry of Health and Child Care
HIV: Human Immunodeficiency Virus
AIDS: Acquired Immunodeficiency Syndrome
ART: Antiretroviral therapy
PLHIV: People living with HIV
IPITC: Intensified Provider Initiated Testing and Counselling
UNAIDS: Joint United Nations Programme on HIV/AIDS
