# Supplementary figures and images for: District-level strategies to control the HIV epidemic in Zimbabwe: a practical example of precision public health
Source: BMC Res Notes. 2020 Aug 26;13:393. doi: 10.1186/s13104-020-05234-8 (PMC7449062; doi:10.1186/s13104-020-05234-8)

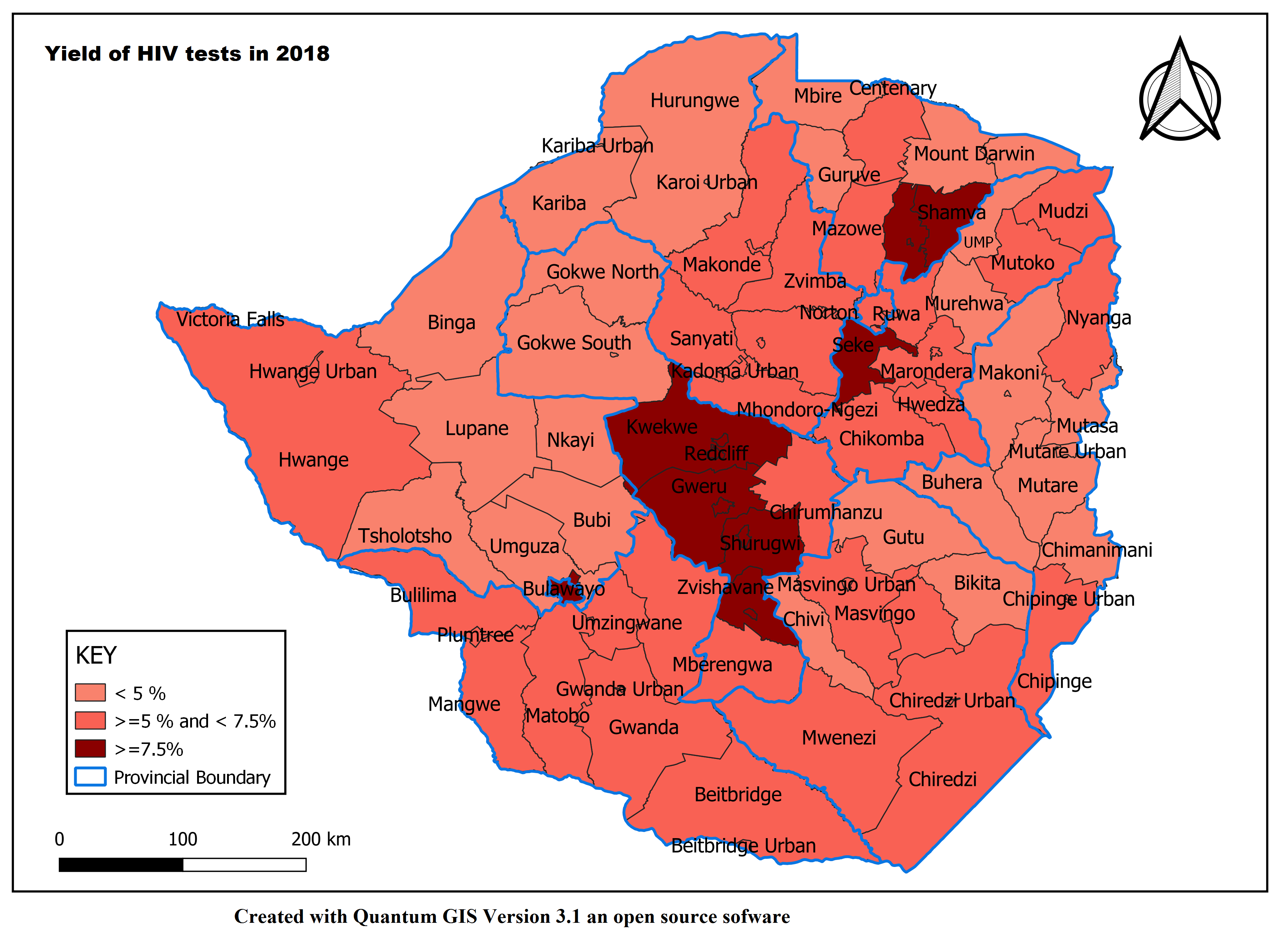

Supplement: Supplementary file 1 — Additional file 1: Figure S1. Yield per district among HIV tests performed in 2018 in Zimbabwe. [file 13104_2020_5234_MOESM1_ESM.png]
